# The genome sequence of the Yellow-dotted Stilt, *Euspilapteryx auroguttella *Stephens, 1835

**DOI:** 10.12688/wellcomeopenres.21284.1

**Published:** 2024-04-24

**Authors:** Douglas Boyes, David C. Lees, Ian Sims, Dominic Phillips, Clare Boyes, University of Oxford and Wytham Woods Genome Acquisition Lab, Natural History Museum Genome Acquisition Lab, Darwin Tree of Life Barcoding collective, Wellcome Sanger Institute Tree of Life Management, Samples and Laboratory team, Wellcome Sanger Institute Scientific Operations: Sequencing Operations, Wellcome Sanger Institute Tree of Life Core Informatics team, Tree of Life Core Informatics collective, Darwin Tree of Life Consortium

**Affiliations:** 1 UK Centre for Ecology & Hydrology, Wallingford, England, UK; 2 Natural History Museum, London, England, UK; 3 Syngenta International Research Station, Jealott’s Hill, Berkshire, UK; 4 Independent resercher, Welshpool, Wales, UK

**Keywords:** Euspilapteryx auroguttella, Yelllow-dotted Stilt, genome sequence, chromosomal, Lepidoptera

## Abstract

We present a genome assembly from an individual male *Euspilapteryx auroguttella* (the Yellow-dotted Stilt; Arthropoda; Insecta; Lepidoptera; Gracillariidae). The genome sequence is 331.9 megabases in span. Most of the assembly is scaffolded into 30 chromosomal pseudomolecules, including the Z sex chromosome. The mitochondrial genome has also been assembled and is 16.94 kilobases in length.

## Species taxonomy

Eukaryota; Opisthokonta; Metazoa; Eumetazoa; Bilateria; Protostomia; Ecdysozoa; Panarthropoda; Arthropoda; Mandibulata; Pancrustacea; Hexapoda; Insecta; Dicondylia; Pterygota; Neoptera; Endopterygota; Amphiesmenoptera; Lepidoptera; Glossata; Neolepidoptera; Heteroneura; Ditrysia; Tineoidea; Gracillariidae; Gracillariinae; *Euspilapteryx*; *Euspilapteryx auroguttella* Stephens, 1835 (NCBI:txid1594449).

## Background

*Euspilapteryx auroguttella,* the Yellow-dotted Stilt ( Sterling *et al.*, 2023), previously also known in Britain as the Gold-dot Slender, is a tiny leaf-mining micro-moth in the family Gracillariidae. This family is unusual amongst the leaf-miners because it exhibits hypermetamorphosis: the first instar larvae are flattened and their mouth faces forwards as an adaptation for living inside the leaves of their host plant; in later instars, the larvae develop legs and a rounded head with a downwardly directed mouth. These later instars feed on the outside of the leaves ( Triberti & Braggio, 2011).

The moth is common in England with a patchier distribution in the rest of the United Kingdom. It is also found in western and central Europe with scattered records as far east as central Russia ( GBIF Secretariat, 2024). The adult (forewing length 4.5–5 mm) has dark metallic grey forewings with a series of orange spots along the wings. The antennae are dark with white tips. Like other moths in the family Gracillariidae it rests with its body held in an incline with the head higher than the abdomen ( Sterling *et al*., 2023: Plate 8). The adult moth is bivoltine flying between April and October, peaking in June and August in coppiced woodland and grassland ( Heath & Emmet, 1985; Sterling *et al*., 2023). The eggs are laid under the leaves of St John’s Wort, *Hypericum* spp, (full species list in Lepiforum (2024)) and the first-instar larvae form a very twisted ‘gallery’ along the leaf. This is later obscured by a blotch on the leaves as the second larval instar feeds. The leaf becomes distorted and the later instars feed on the outside of the leaf which is spun into a downwardly pointed cone. The larvae pupate in a folded leaf on the ground ( Langmaid *et al.*, 2018). The mines and a later instar larva were first illustrated by Stainton (1864). No *Wolbachia* infection was found in this species in the study of Gutzwiller *et al.* (2015) in their study of “green island” formation which is a frequent endobiotic association in Gracillariidae leaf miners.

*Euspilapteryx auroguttella* forms a single cluster on BOLD (BOLD:AAD7434) (05/03/2024) which is isolated to other gracillariids (over 8.1 % pairwise divergent to the nearest species of *Caloptilia* Hübner, [1825] ( Lopez-Vaamonde *et al.*, 2021). The COI-5P region of the sequenced mitogenome (OX637671.1) shows no difference to many European exemplars of *E. auroguttella* on BOLD. The species was recovered as sister to *Eucalybites aureola* Kumata, 1982, also monobasic, in the up to 22 gene phylogeny of Kawahara *et al.* ( 2017: Figs 2, 3), in which, however, the monophyly of *Caloptilia*, also final instar leaf rollers, was not recovered.

The genome of *Euspilapteryx auroguttella* was sequenced as part of the Darwin Tree of Life Project, a collaborative effort to sequence all named eukaryotic species in the Atlantic Archipelago of Britain and Ireland. This genome sequence will be aid research into hypermetamorphosis in moths.

## Genome sequence report

**Figure 1.**
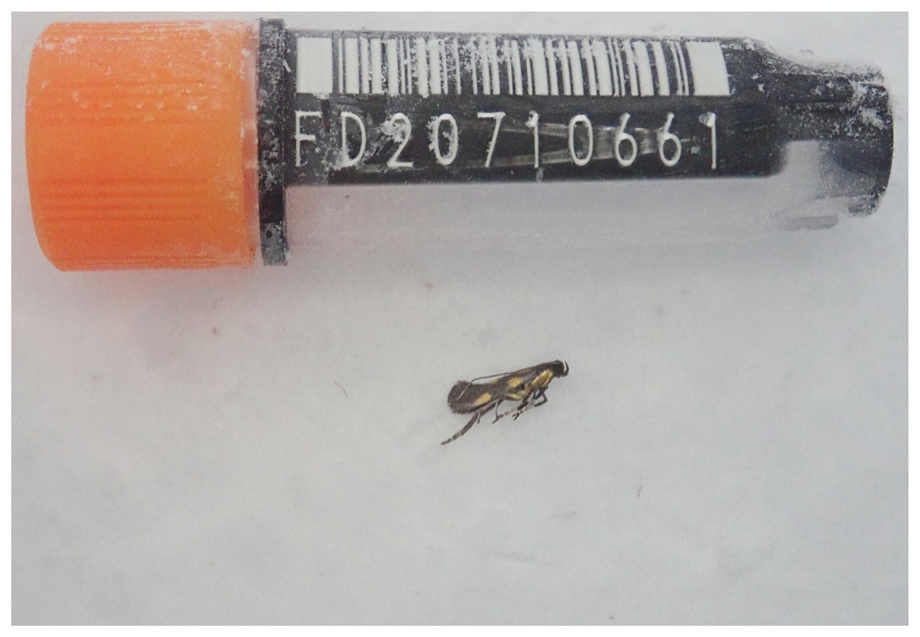
Photograph of the *Euspilapteryx auroguttella* (ilEusAuro2) specimen used for genome sequencing.

The genome was sequenced from a male *Euspilapteryx auroguttella* ( Figure 1) collected from Wytham Woods, Oxfordshire, UK (51.77, –1.34). A total of 74-fold coverage in Pacific Biosciences single-molecule HiFi long reads was generated. Primary assembly contigs were scaffolded with chromosome conformation Hi-C data. Manual assembly curation corrected 41 missing joins or mis-joins and removed 9 haplotypic duplications, reducing the assembly length by 0.35% and the scaffold number by 14.17%, and also reducing the scaffold N50 by 0.50%.

**Figure 2.**
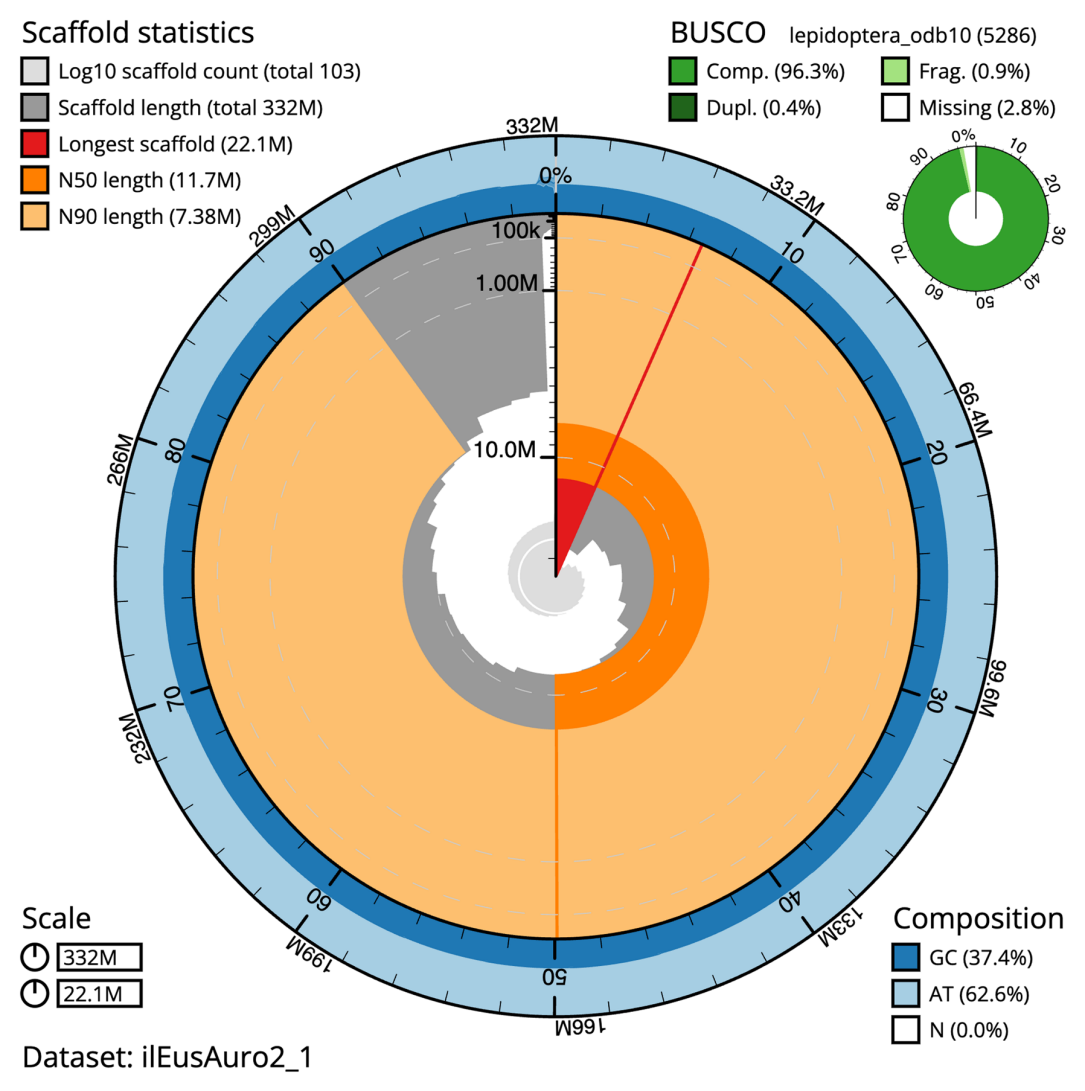
Genome assembly of *Euspilapteryx auroguttella*, ilEusAuro2.1: metrics. The BlobToolKit snail plot shows N50 metrics and BUSCO gene completeness. The main plot is divided into 1,000 size-ordered bins around the circumference with each bin representing 0.1% of the 331,914,766 bp assembly. The distribution of scaffold lengths is shown in dark grey with the plot radius scaled to the longest scaffold present in the assembly (22,118,606 bp, shown in red). Orange and pale-orange arcs show the N50 and N90 scaffold lengths (11,650,979 and 7,375,079 bp), respectively. The pale grey spiral shows the cumulative scaffold count on a log scale with white scale lines showing successive orders of magnitude. The blue and pale-blue area around the outside of the plot shows the distribution of GC, AT and N percentages in the same bins as the inner plot. A summary of complete, fragmented, duplicated and missing BUSCO genes in the lepidoptera_odb10 set is shown in the top right. An interactive version of this figure is available at https://blobtoolkit.genomehubs.org/view/ilEusAuro2_1/dataset/ilEusAuro2_1/snail.

**Figure 3.**
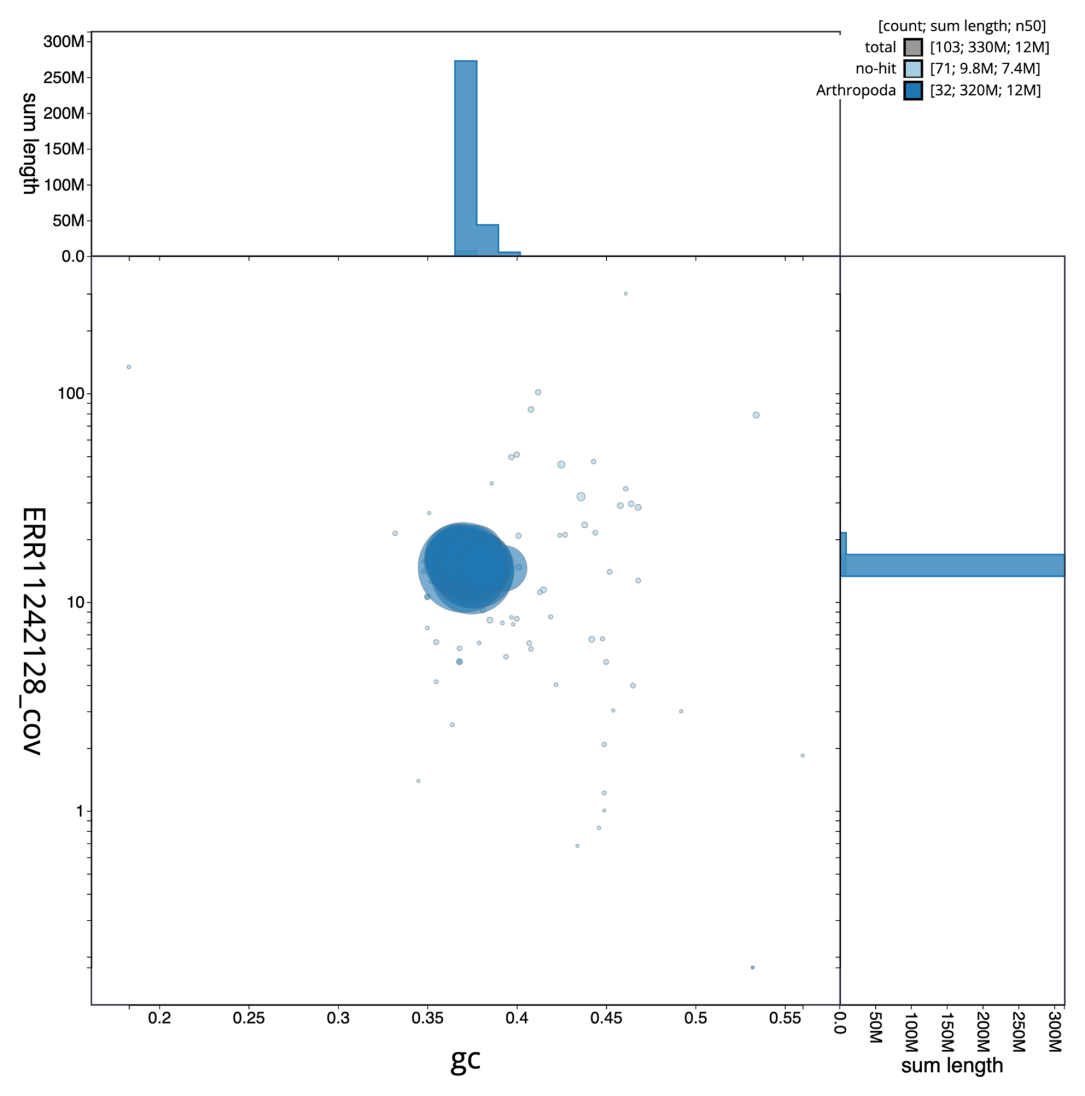
Genome assembly of *Euspilapteryx auroguttella*, ilEusAuro2.1: BlobToolKit GC-coverage plot. Sequences are coloured by phylum. Circles are sized in proportion to sequence length. Histograms show the distribution of sequence length sum along each axis. An interactive version of this figure is available at https://blobtoolkit.genomehubs.org/view/ilEusAuro2_1/dataset/ilEusAuro2_1/blob.

**Figure 4.**
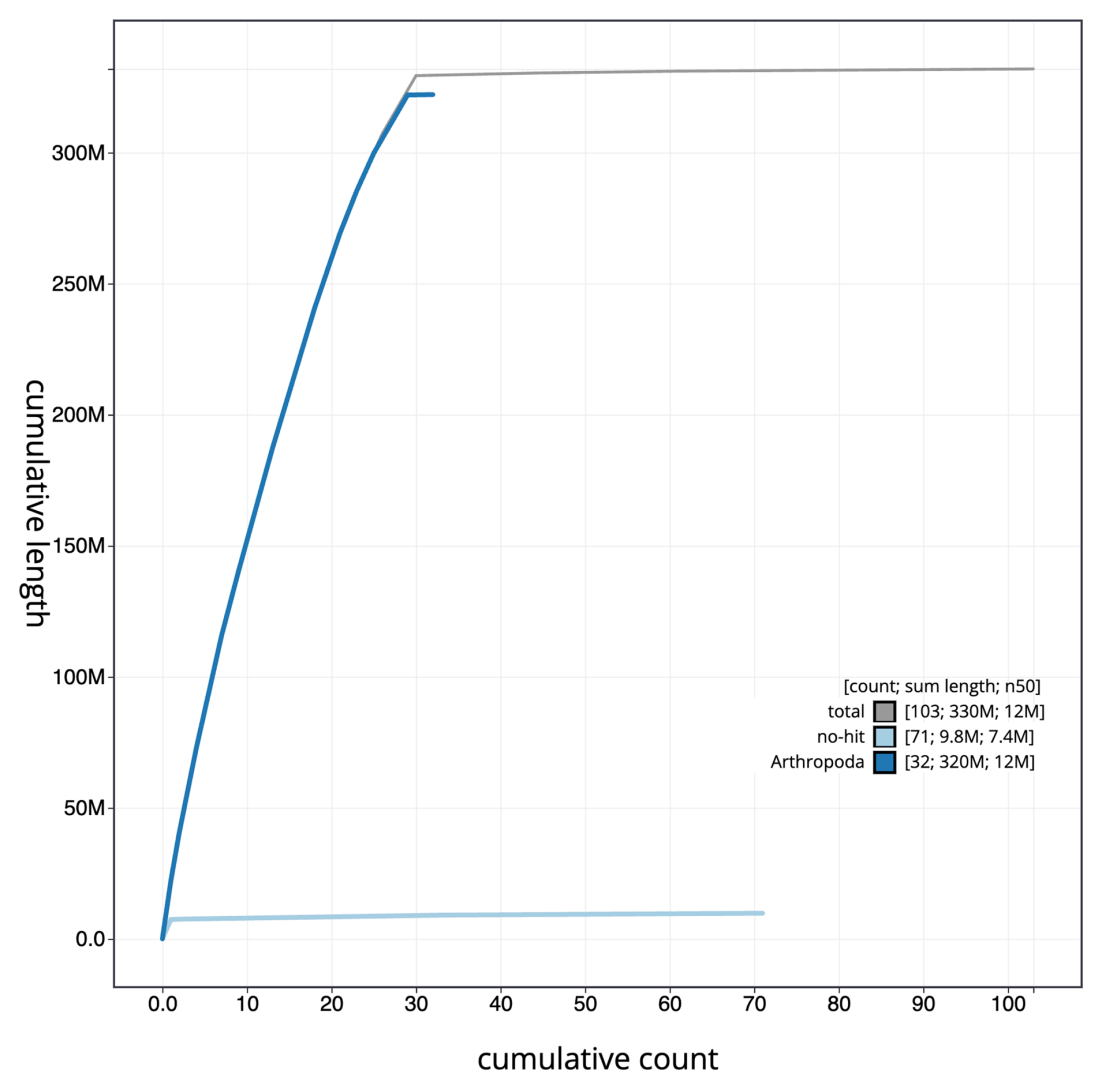
Genome assembly of *Euspilapteryx auroguttella*, ilEusAuro2.1: BlobToolKit cumulative sequence plot. The grey line shows cumulative length for all sequences. Coloured lines show cumulative lengths of sequences assigned to each phylum using the buscogenes taxrule. An interactive version of this figure is available at https://blobtoolkit.genomehubs.org/view/ilEusAuro2_1/dataset/ilEusAuro2_1/cumulative.

**Figure 5.**
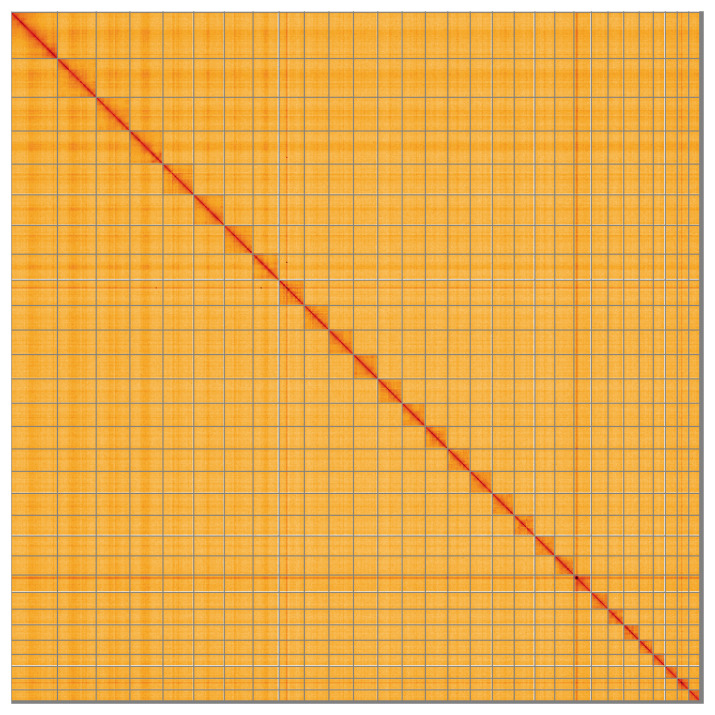
Genome assembly of *Euspilapteryx auroguttella*, ilEusAuro2.1: Hi-C contact map of the ilEusAuro2.1 assembly, visualised using HiGlass. Chromosomes are shown in order of size from left to right and top to bottom. An interactive version of this figure may be viewed at https://genome-note-higlass.tol.sanger.ac.uk/l/?d=D2WioQoKTxeVhnV9O1oKSg.

The final assembly has a total length of 331.9 Mb in 102 sequence scaffolds with a scaffold N50 of 11.7 Mb ( Table 1). The snail plot in Figure 2 provides a summary of the assembly statistics, while the distribution of assembly scaffolds on GC proportion and coverage is shown in Figure 3. The cumulative assembly plot in Figure 4 shows curves for subsets of scaffolds assigned to different phyla. Most (99.23%) of the assembly sequence was assigned to 30 chromosomal-level scaffolds, representing 29 autosomes and the Z sex chromosome. Chromosome-scale scaffolds confirmed by the Hi-C data are named in order of size ( Figure 5; Table 2). The Z chromosome assignment was based on synteny with *Tinea pellionella* (GCA_948150575.1). While not fully phased, the assembly deposited is of one haplotype. Contigs corresponding to the second haplotype have also been deposited. The mitochondrial genome was also assembled and can be found as a contig within the multifasta file of the genome submission.

**Table 1.** Genome data for *Euspilapteryx auroguttella*, ilEusAuro2.1.

Project accession data		
Assembly identifier	ilEusAuro2.1	
Species	*Euspilapteryx auroguttella*	
Specimen	ilEusAuro2	
NCBI taxonomy ID	1594449	
BioProject	PRJEB61339	
BioSample ID	SAMEA10979071	
Isolate information	ilEusAuro2: whole organism (DNA sequencing) ilEusAuro4: whole organism (Hi-C sequencing) ilEusAuro5: whole organism (RNA sequencing)	
Assembly metrics *		*Benchmark*
Consensus quality (QV)	58.6	*≥ 50*
*k*-mer completeness	100.0%	*≥ 95%*
BUSCO **	C:96.3%[S:95.9%,D:0.4%], F:0.9%,M:2.8%,n:5,286	*C ≥ 95%*
Percentage of assembly mapped to chromosomes	99.23%	*≥ 95%*
Sex chromosomes	Z	*localised homologous pairs*
Organelles	Mitochondrial genome: 16.94 kb	*complete single alleles*
Raw data accessions		
PacificBiosciences SEQUEL II	ERR11242129, ERR11242128	
Hi-C Illumina	ERR11242546	
PolyA RNA-Seq Illumina	ERR12245557	
Genome assembly		
Assembly accession	GCA_951802225.1	
*Accession of alternate haplotype*	GCA_951802235.1	
Span (Mb)	331.9	
Number of contigs	471	
Contig N50 length (Mb)	2.0	
Number of scaffolds	102	
Scaffold N50 length (Mb)	11.7	
Longest scaffold (Mb)	22.12	

* Assembly metric benchmarks are adapted from column VGP-2020 of “Table 1: Proposed standards and metrics for defining genome assembly quality” from Rhie *et al.* (2021). ** BUSCO scores based on the lepidoptera_odb10 BUSCO set using version 5.3.2. C = complete [S = single copy, D = duplicated], F = fragmented, M = missing, n = number of orthologues in comparison. A full set of BUSCO scores is available at https://blobtoolkit.genomehubs.org/view/ilEusAuro2_1/dataset/ilEusAuro2_1/busco.

**Table 2.** Chromosomal pseudomolecules in the genome assembly of *Euspilapteryx auroguttella*, ilEusAuro2.

INSDC accession	Chromosome	Length (Mb)	GC%
OX637642.1	1	18.51	37.5
OX637643.1	2	16.14	37.5
OX637644.1	3	15.84	37.5
OX637645.1	4	14.7	37.0
OX637646.1	5	14.61	37.5
OX637647.1	6	13.79	37.0
OX637648.1	7	12.43	37.0
OX637649.1	8	12.09	36.5
OX637650.1	9	11.77	36.5
OX637651.1	10	11.71	37.5
OX637652.1	11	11.65	37.0
OX637653.1	12	11.64	37.0
OX637654.1	13	11.11	37.5
OX637655.1	14	10.76	37.0
OX637656.1	15	10.74	37.5
OX637657.1	16	10.57	37.5
OX637658.1	17	10.47	38.0
OX637659.1	18	9.9	37.5
OX637660.1	19	9.51	37.0
OX637661.1	20	9.16	38.0
OX637662.1	21	8.32	37.5
OX637663.1	22	7.99	37.5
OX637664.1	23	7.51	38.0
OX637665.1	24	7.38	37.5
OX637666.1	25	6.76	37.5
OX637667.1	26	5.74	38.0
OX637668.1	27	5.72	38.0
OX637669.1	28	5.51	39.5
OX637670.1	29	5.24	38.0
OX637641.1	Z	22.12	37.0
OX637671.1	MT	0.02	18.5

The estimated Quality Value (QV) of the final assembly is 58.6 with *k*-mer completeness of 100.0%, and the assembly has a BUSCO v5.3.2 completeness of 96.3% (single = 95.9%, duplicated = 0.4%), using the lepidoptera_odb10 reference set ( *n* = 5,286).

Metadata for specimens, barcode results, spectra estimates, sequencing runs, contaminants and pre-curation assembly statistics are given at https://links.tol.sanger.ac.uk/species/1594449.

## Methods

### Sample acquisition and nucleic acid extraction

A male *Euspilapteryx auroguttella* (specimen ID Ox001813, ToLID ilEusAuro2) was collected from Wytham Woods, Oxfordshire (biological vice-county Berkshire), UK (latitude 51.77, longitude –1.34) on 2021-07-24 using a light trap. The specimen was collected and identified by Douglas Boyes (University of Oxford) and preserved on dry ice.

The specimen used for Hi-C sequencing (specimen ID NHMUK013805989, ToLID ilEusAuro4) was collected from Hartslock National Nature Reserve, England, UK (latitude 51.51, longitude –1.11) on 2021-07-29, also using a light trap. The specimen was collected by Ian Sims (independent researcher) and identified by David Lees (Natural History Museum) and Ian Sims. A third specimen, used for RNA sequencing (specimen ID NHMUK015050629, ToLID ilEusAuro5), was collected from the Nature Scot visitor centre, Beinn Eighe, Scotland, UK (latitude 57.61, longitude –5.31) on 2022-04-26, using an aerial net. The specimen was collected by David Lees and Dominic Phillips (Natural History Museum) and identified by David Lees. Both of these specimens were preserved by dry freezing at –80°C.

The workflow for high molecular weight (HMW) DNA extraction at the Wellcome Sanger Institute (WSI) includes a sequence of core procedures: sample preparation; sample homogenisation, DNA extraction, fragmentation, and clean-up. The sample was prepared for DNA extraction at the WSI Tree of Life Core Laboratory: the ilEusAuro2 sample was weighed and dissected on dry ice ( Jay *et al.*, 2023). Tissue from the whole organism was homogenised using a PowerMasher II tissue disruptor ( Denton *et al.*, 2023a).

HMW DNA was extracted using the Automated MagAttract v1 protocol ( Sheerin *et al.*, 2023). DNA was sheared into an average fragment size of 12–20 kb in a Megaruptor 3 system with speed setting 30 ( Todorovic *et al.*, 2023). Sheared DNA was purified by solid-phase reversible immobilisation ( Strickland *et al.*, 2023): in brief, the method employs a 1.8X ratio of AMPure PB beads to sample to eliminate shorter fragments and concentrate the DNA. The concentration of the sheared and purified DNA was assessed using a Nanodrop spectrophotometer and Qubit Fluorometer and Qubit dsDNA High Sensitivity Assay kit. Fragment size distribution was evaluated by running the sample on the FemtoPulse system.

RNA was extracted from whole organism tissue of ilEusAuro5 in the Tree of Life Laboratory at the WSI using the RNA Extraction: Automated MagMax™ *mir*Vana protocol ( do Amaral *et al.*, 2023). The RNA concentration was assessed using a Nanodrop spectrophotometer and a Qubit Fluorometer using the Qubit RNA Broad-Range Assay kit. Analysis of the integrity of the RNA was done using the Agilent RNA 6000 Pico Kit and Eukaryotic Total RNA assay.

Protocols developed by the WSI Tree of Life laboratory are publicly available on protocols.io ( Denton *et al.*, 2023b).

### Sequencing

Pacific Biosciences HiFi circular consensus DNA sequencing libraries were constructed according to the manufacturers’ instructions. Poly(A) RNA-Seq libraries were constructed using the NEB Ultra II RNA Library Prep kit. DNA and RNA sequencing was performed by the Scientific Operations core at the WSI on Pacific Biosciences SEQUEL II (HiFi) and Illumina NovaSeq 6000 (RNA-Seq) instruments. Hi-C data were also generated from whole organism tissue of ilEusAuro4 using the Arima2 kit and sequenced on the Illumina NovaSeq 6000 instrument.

### Genome assembly, curation and evaluation

Assembly was carried out with Hifiasm ( Cheng *et al.*, 2021) and haplotypic duplication was identified and removed with purge_dups ( Guan *et al.*, 2020). The assembly was then scaffolded with Hi-C data ( Rao *et al.*, 2014) using YaHS ( Zhou *et al.*, 2023). The assembly was checked for contamination and corrected as described previously ( Howe *et al.*, 2021). Manual curation was performed using HiGlass ( Kerpedjiev *et al.*, 2018) and PretextView ( Harry, 2022). The mitochondrial genome was assembled using MitoHiFi ( Uliano-Silva *et al.*, 2023), which runs MitoFinder ( Allio *et al.*, 2020) or MITOS ( Bernt *et al.*, 2013) and uses these annotations to select the final mitochondrial contig and to ensure the general quality of the sequence.

A Hi-C map for the final assembly was produced using bwa-mem2 ( Vasimuddin *et al.*, 2019) in the Cooler file format ( Abdennur & Mirny, 2020). To assess the assembly metrics, the *k*-mer completeness and QV consensus quality values were calculated in Merqury ( Rhie *et al.*, 2020). This work was done using Nextflow ( Di Tommaso *et al.*, 2017) DSL2 pipelines “sanger-tol/readmapping” ( Surana *et al.*, 2023a) and “sanger-tol/genomenote” ( Surana *et al.*, 2023b). The genome was analysed within the BlobToolKit environment ( Challis *et al.*, 2020) and BUSCO scores ( Manni *et al.*, 2021; Simão *et al.*, 2015) were calculated.

Table 3 contains a list of relevant software tool versions and sources.

**Table 3.** Software tools: versions and sources.

Software tool	Version	Source
BlobToolKit	4.2.1	https://github.com/blobtoolkit/blobtoolkit
BUSCO	5.3.2	https://gitlab.com/ezlab/busco
Hifiasm	0.16.1-r375	https://github.com/chhylp123/hifiasm
HiGlass	1.11.6	https://github.com/higlass/higlass
Merqury	MerquryFK	https://github.com/thegenemyers/MERQURY.FK
MitoHiFi	3	https://github.com/marcelauliano/MitoHiFi
PretextView	0.2	https://github.com/wtsi-hpag/PretextView
purge_dups	1.2.3	https://github.com/dfguan/purge_dups
sanger-tol/genomenote	v1.0	https://github.com/sanger-tol/genomenote
sanger-tol/readmapping	1.1.0	https://github.com/sanger-tol/readmapping/tree/1.1.0
YaHS	1.2a.2	https://github.com/c-zhou/yahs

### Wellcome Sanger Institute – Legal and Governance

The materials that have contributed to this genome note have been supplied by a Darwin Tree of Life Partner. The submission of materials by a Darwin Tree of Life Partner is subject to the **‘Darwin Tree of Life Project Sampling Code of Practice’**, which can be found in full on the Darwin Tree of Life website here. By agreeing with and signing up to the Sampling Code of Practice, the Darwin Tree of Life Partner agrees they will meet the legal and ethical requirements and standards set out within this document in respect of all samples acquired for, and supplied to, the Darwin Tree of Life Project. 

Further, the Wellcome Sanger Institute employs a process whereby due diligence is carried out proportionate to the nature of the materials themselves, and the circumstances under which they have been/are to be collected and provided for use. The purpose of this is to address and mitigate any potential legal and/or ethical implications of receipt and use of the materials as part of the research project, and to ensure that in doing so we align with best practice wherever possible. The overarching areas of consideration are:

•     Ethical review of provenance and sourcing of the material

•     Legality of collection, transfer and use (national and international)

Each transfer of samples is further undertaken according to a Research Collaboration Agreement or Material Transfer Agreement entered into by the Darwin Tree of Life Partner, Genome Research Limited (operating as the Wellcome Sanger Institute), and in some circumstances other Darwin Tree of Life collaborators.

## Data Availability

European Nucleotide Archive: *Euspilapteryx auroguttella* (gold-dot slender). Accession number PRJEB61339; https://identifiers.org/ena.embl/PRJEB61339 ( Wellcome Sanger Institute, 2023). The genome sequence is released openly for reuse. The *Euspilapteryx auroguttella* genome sequencing initiative is part of the Darwin Tree of Life (DToL) project. All raw sequence data and the assembly have been deposited in INSDC databases. The genome will be annotated using available RNA-Seq data and presented through the Ensembl pipeline at the European Bioinformatics Institute. Raw data and assembly accession identifiers are reported in Table 1.

## Author information

Members of the University of Oxford and Wytham Woods Genome Acquisition Lab are listed here: https://doi.org/10.5281/zenodo.7125292.

Members of the Natural History Museum Genome Acquisition Lab are listed here: https://doi.org/10.5281/zenodo.7139035.

Members of the Darwin Tree of Life Barcoding collective are listed here: https://doi.org/10.5281/zenodo.4893703.

Members of the Wellcome Sanger Institute Tree of Life Management, Samples and Laboratory team are listed here: https://doi.org/10.5281/zenodo.10066175.

Members of Wellcome Sanger Institute Scientific Operations: Sequencing Operations are listed here: https://doi.org/10.5281/zenodo.10043364.

Members of the Wellcome Sanger Institute Tree of Life Core Informatics team are listed here: https://doi.org/10.5281/zenodo.10066637.

Members of the Tree of Life Core Informatics collective are listed here: https://doi.org/10.5281/zenodo.5013541.

Members of the Darwin Tree of Life Consortium are listed here: https://doi.org/10.5281/zenodo.4783558.

## References

[ref-1] Abdennur N Mirny LA : Cooler: scalable storage for Hi-C data and other genomically labeled arrays. *Bioinformatics.* 2020;36(1):311–316. 10.1093/bioinformatics/btz540 31290943 PMC8205516

[ref-2] Allio R Schomaker-Bastos A Romiguier J : MitoFinder: efficient automated large-scale extraction of mitogenomic data in target enrichment phylogenomics. *Mol Ecol Resour.* 2020;20(4):892–905. 10.1111/1755-0998.13160 32243090 PMC7497042

[ref-3] Bernt M Donath A Jühling F : MITOS: Improved *de novo* metazoan mitochondrial genome annotation. *Mol Phylogenet Evol.* 2013;69(2):313–319. 10.1016/j.ympev.2012.08.023 22982435

[ref-4] Challis R Richards E Rajan J : BlobToolKit – Interactive quality assessment of genome assemblies. *G3 (Bethesda).* 2020;10(4):1361–1374. 10.1534/g3.119.400908 32071071 PMC7144090

[ref-5] Cheng H Concepcion GT Feng X : Haplotype-resolved *de novo* assembly using phased assembly graphs with hifiasm. *Nat Methods.* 2021;18(2):170–175. 10.1038/s41592-020-01056-5 33526886 PMC7961889

[ref-6] Denton A Oatley G Cornwell C : Sanger Tree of Life Sample homogenisation: PowerMash. *protocols.io.* 2023a. 10.17504/protocols.io.5qpvo3r19v4o/v1

[ref-7] Denton A Yatsenko H Jay J : Sanger Tree of Life wet laboratory protocol collection V.1. *protocols.io.* 2023b. 10.17504/protocols.io.8epv5xxy6g1b/v1

[ref-8] Di Tommaso P Chatzou M Floden EW : Nextflow enables reproducible computational workflows. *Nat Biotechnol.* 2017;35(4):316–319. 10.1038/nbt.3820 28398311

[ref-9] do Amaral RJV Bates A Denton A : Sanger Tree of Life RNA extraction: automated MagMax ^TM^ mirVana. *protocols.io.* 2023. 10.17504/protocols.io.6qpvr36n3vmk/v1

[ref-10] GBIF Secretariat: *Euspilapteryx auroguttella* Stephens, 1835. *GBIF Backbone Taxonomy.* 2024; [Accessed 1 March 2024]. Reference Source

[ref-11] Guan D McCarthy SA Wood J : Identifying and removing haplotypic duplication in primary genome assemblies. *Bioinformatics.* 2020;36(9):2896–2898. 10.1093/bioinformatics/btaa025 31971576 PMC7203741

[ref-12] Gutzwiller F Dedeine F Kaiser W : Correlation between the Green-Island phenotype and *Wolbachia* infections during the evolutionary diversification of Gracillariidae leaf-mining moths. *Ecol Evol.* 2015;5(18):4049–4062. 10.1002/ece3.1580 26442762 PMC4588643

[ref-13] Harry E : PretextView (Paired REad TEXTure Viewer): a desktop application for viewing pretext contact maps. 2022; [Accessed 19 October 2022]. Reference Source

[ref-14] Heath J Emmet AM : The moths and butterflies of Great Britain and Ireland cossidae - heliodinidae.Colchester: Harley Books,1985.

[ref-15] Howe K Chow W Collins J : Significantly improving the quality of genome assemblies through curation. *GigaScience.* Oxford University Press,2021;10(1): giaa153. 10.1093/gigascience/giaa153 33420778 PMC7794651

[ref-16] Jay J Yatsenko H Narváez-Gómez JP : Sanger Tree of Life Sample preparation: triage and dissection. *protocols.io.* 2023. 10.17504/protocols.io.x54v9prmqg3e/v1

[ref-17] Kawahara AY Plotkin D Ohshima I : A molecular phylogeny and revised higher-level classification for the leaf-mining moth family Gracillariidae and its implications for larval host-use evolution. *Syst Entomol.* 2017;42(1):60–81. 10.1111/syen.12210

[ref-18] Kerpedjiev P Abdennur N Lekschas F : HiGlass: web-based visual exploration and analysis of genome interaction maps. *Genome Biol.* 2018;19(1): 125. 10.1186/s13059-018-1486-1 30143029 PMC6109259

[ref-19] Langmaid JR Palmer S Young MR : A field guide to the smaller moths of Great Britain and Ireland.3rd ed. British Entomological and Natural History Society,2018. Reference Source

[ref-20] Lepiforum: *Euspilapteryx auroguttella* . 2024; [Accessed 13 March 2024]. Reference Source

[ref-21] Lopez-Vaamonde C Kirichenko N Cama A : Evaluating DNA barcoding for species identification and discovery in European Gracillariid moths. *Front Ecology and Evol.* 2021;9: 626752. 10.3389/fevo.2021.626752

[ref-22] Manni M Berkeley MR Seppey M : BUSCO update: novel and streamlined workflows along with broader and deeper phylogenetic coverage for scoring of eukaryotic, prokaryotic, and viral genomes. *Mol Biol Evol.* 2021;38(10):4647–4654. 10.1093/molbev/msab199 34320186 PMC8476166

[ref-23] Rao SSP Huntley MH Durand NC : A 3D map of the human genome at kilobase resolution reveals principles of chromatin looping. *Cell.* 2014;159(7):1665–1680. 10.1016/j.cell.2014.11.021 25497547 PMC5635824

[ref-24] Rhie A McCarthy SA Fedrigo O : Towards complete and error-free genome assemblies of all vertebrate species. *Nature.* 2021;592(7856):737–746. 10.1038/s41586-021-03451-0 33911273 PMC8081667

[ref-25] Rhie A Walenz BP Koren S : Merqury: reference-free quality, completeness, and phasing assessment for genome assemblies. *Genome Biol.* 2020;21(1): 245. 10.1186/s13059-020-02134-9 32928274 PMC7488777

[ref-26] Sheerin E Sampaio F Oatley G : Sanger Tree of Life HMW DNA extraction: automated MagAttract v.1. *protocols.io.* 2023. 10.17504/protocols.io.x54v9p2z1g3e/v1

[ref-27] Simão FA Waterhouse RM Ioannidis P : BUSCO: assessing genome assembly and annotation completeness with single-copy orthologs. *Bioinformatics.* 2015;31(19):3210–3212. 10.1093/bioinformatics/btv351 26059717

[ref-28] Stainton HT : The natural history of the Tineina, 8 (I-IX): Gracilaria pl. I-V.London: John van Voorst,1864. Reference Source

[ref-29] Sterling P Parsons M Lewington R : Field guide to the micro-moths of Great Britain and Ireland.London: Bloomsbury Publishing,2023. Reference Source

[ref-30] Strickland M Cornwell C Howard C : Sanger Tree of Life fragmented DNA clean up: manual SPRI. *protocols.io.* 2023. 10.17504/protocols.io.kxygx3y1dg8j/v1

[ref-31] Surana P Muffato M Qi G : Sanger-tol/readmapping: sanger-tol/readmapping v1.1.0 - Hebridean Black (1.1.0). *Zenodo*. 2023a. 10.5281/zenodo.7755669

[ref-32] Surana P Muffato M Sadasivan Baby C : Sanger-tol/genomenote (v1.0.dev). *Zenodo*. 2023b. 10.5281/zenodo.6785935

[ref-33] Todorovic M Sampaio F Howard C : Sanger Tree of Life HMW DNA fragmentation: Diagenode Megaruptor ^®^3 for PacBio HiFi. *protocols.io.* 2023. 10.17504/protocols.io.8epv5x2zjg1b/v1

[ref-34] Triberti P Braggio S : Remarks on some families of leaf-mining microlepidoptera from Central-Southern Sardinia, with some ecological considerations (Lepidoptera: Nepticulidae, Bucculatricidae, Gracillariidae). *Conservazione Habitat Invertebrati.* 2011;5:767–781. Reference Source

[ref-35] Uliano-Silva M Ferreira JGRN Krasheninnikova K : MitoHiFi: a python pipeline for mitochondrial genome assembly from PacBio high fidelity reads. *BMC Bioinformatics.* 2023;24(1): 288. 10.1186/s12859-023-05385-y 37464285 PMC10354987

[ref-36] Vasimuddin M Misra S Li H : Efficient architecture-aware acceleration of BWA-MEM for multicore systems.In: *2019 IEEE International Parallel and Distributed Processing Symposium (IPDPS).*IEEE,2019;314–324. 10.1109/IPDPS.2019.00041

[ref-37] Wellcome Sanger Institute: The genome sequence of the Yellow-dotted Stilt, *Euspilapteryx auroguttella* Stephens, 1835. European Nucleotide Archive.[dataset], accession number PRJEB61339,2023.

[ref-38] Zhou C McCarthy SA Durbin R : YaHS: yet another Hi-C scaffolding tool. *Bioinformatics.* 2023;39(1): btac808. 10.1093/bioinformatics/btac808 36525368 PMC9848053

