# Supplementary material for: The genome sequence of the Yellow-dotted Stilt, Euspilapteryx auroguttella Stephens, 1835
Source: Wellcome Open Res. 2024 Apr 24;9:226. [Version 1] doi: 10.12688/wellcomeopenres.21284.1 (PMC11459120; doi:10.12688/wellcomeopenres.21284.1)
Supplement: Peer review history

# Reviewer report: Reviewer response for version 1

**DOI:** 10.21956/wellcomeopenres.23540.r101577

**Published:** 2024-10-07

**Authors:** Marta Coronado-Zamora

**Affiliations:** 1 Botanical Institute of Barcelona, Barcelona, Catalonia, Spain

This data note describes a high-quality assembly of the Yellow-dotted Stilt, using the most advanced sequencing and assembly methods. The genome data are available at ENA and data is accessible.

**Comments:**

- RNA-seq is also generated for this species, however, this is not mentioned in the Results section. 
- Introduce the sex determination of this specifies could be useful (it's only mentioned in the abstract).
- Regarding the other two specimens used for Hi-C and RNA-seq: the sex is missing.
- Also, which tissue was used? or was whole-body? Because in the project site
  ( https://tolqc.cog.sanger.ac.uk/darwin/insects/Euspilapteryx_auroguttella/) it is mentioned abdomen and thorax, but this information is not in the report.
- Specify that the numbers after mentioning the collection site in the Genome sequence report section refers to longitude and latitude, as done in the methods.
- "bwa-mem2" is missing from Table 3

Are sufficient details of methods and materials provided to allow replication by others?

Yes

Is the rationale for creating the dataset(s) clearly described?

Yes

Are the datasets clearly presented in a useable and accessible format?

Yes

Are the protocols appropriate and is the work technically sound?

Yes

Reviewer Expertise:

bioinformatics, NGS analysis, population genetics, epigenetics, transposable elements, adaptation

I confirm that I have read this submission and believe that I have an appropriate level of expertise to confirm that it is of an acceptable scientific standard.

# Reviewer report: Reviewer response for version 1

**DOI:** 10.21956/wellcomeopenres.23540.r93532

**Published:** 2024-08-29

**Authors:** Michal Rindoš

**Affiliations:** 1 University of South Bohemia, Ceske Budejovice, Czech Republic

The *Euspilapteryx auroguttella* genome note contains all the important information about this species, its biology and distribution.

 What I would perhaps welcome is a better specimen photo - in the case of the Microlepidoptera perhaps the second photography with a zoomed specimen could be added and listed as Fig.1 A&B.

Are sufficient details of methods and materials provided to allow replication by others?

Yes

Is the rationale for creating the dataset(s) clearly described?

Yes

Are the datasets clearly presented in a useable and accessible format?

Yes

Are the protocols appropriate and is the work technically sound?

Yes

Reviewer Expertise:

Biology, Biogeogegraphy and Systematics of Lepidoptera

I confirm that I have read this submission and believe that I have an appropriate level of expertise to confirm that it is of an acceptable scientific standard.
